# Identification and prevalence of *in vivo*-induced genes in enterohaemorrhagic *Escherichia coli*

**DOI:** 10.1080/21505594.2019.1582976

**Published:** 2019-03-16

**Authors:** Marion Gardette, Simon Le Hello, Patricia Mariani-Kurkdjian, Laetitia Fabre, François Gravey, Annie Garrivier, Estelle Loukiadis, Grégory Jubelin

**Affiliations:** a UCA, INRA, UMR454 MEDIS, Clermont-Ferrand, France; b Laboratoire d’écologie microbienne de Lyon, Université de Lyon, CNRS, INRA, UCBL, VetAgro Sup, Marcy l’Etoile, France; c Centre de Référence National des Escherichia coli, Shigella et Salmonella, Institut Pasteur, Paris, France; d Université de Normandie, EA 2656 GRAM 2.0, UNICAEN, Caen, France; e Service de Microbiologie, Centre National de Référence associé Escherichia coli, Hôpital Robert-Debré, AP-HP, Paris, France; f Laboratoire national de référence des E. coli, Université de Lyon, VetAgro Sup, Marcy l’Etoile, France

**Keywords:** EHEC, intestinal pathogen, virulence, metabolism, stress response, gene prevalence, RIVET

## Abstract

Enterohaemorrhagic *Escherichia coli* (EHEC) are food-borne pathogens responsible for bloody diarrhoea and renal failure in humans. While Shiga toxin (Stx) is the cardinal virulence factor of EHEC, its production by *E. coli* is not sufficient to cause disease and many Shiga-toxin producing *E. coli* (STEC) strains have never been implicated in human infection. So far, the pathophysiology of EHEC infection is not fully understood and more knowledge is needed to characterize the “auxiliary” factors that enable a STEC strain to cause disease in humans. In this study, we applied a recombinase-based *in vivo* expression technology (RIVET) to the EHEC reference strain EDL933 in order to identify genes specifically induced during the infectious process, using mouse as an infection model. We identified 31 *in vivo*-induced (*ivi*) genes having functions related to metabolism, stress adaptive response and bacterial virulence or fitness. Eight of the 31 *ivi* genes were found to be heterogeneously distributed in EHEC strains circulating in France these last years. In addition, they are more prevalent in strains from the TOP seven priority serotypes and particularly strains carrying significant virulence determinants such as Stx2 and intimin adhesin. This work sheds further light on bacterial determinants over-expressed *in vivo* during infection that may contribute to the potential of STEC strains to cause disease in humans.

## Introduction

Shiga toxin-producing *Escherichia coli* (STEC) are recognized as an important cause of food-borne disease in humans and cause large outbreaks worldwide. Pathogenic STEC, designated as enterohaemorrhagic *E. coli* (EHEC), are responsible for intestinal disorders, including watery or bloody diarrhoea, that may ultimately evolve to life-threatening diseases such as haemolytic uraemic syndrome (HUS) or thrombotic thrombocytopenic purpura. The two cardinal virulence factors in EHEC are type III secretion system (T3SS) and Shiga toxins (Stx). T3SS, encoded by the Locus of Enterocyte Effacement (LEE), is involved in the formation of attaching and effacing (A/E) lesions on gut mucosa through the injection of speciﬁc effectors within enterocytes [1]. Interactions of bacterial effectors with eukaryotic signal transduction pathways lead to host cytoskeleton reorganization that is characterized by two key markers of A/E lesions: an effacement of microvilli and formation of pedestals beneath adherent EHEC. Among translocated bacterial proteins, Tir is crucial since it acts as a receptor for the adhesin intimin (encoded by gene *eae*), upon its integration into the host cell plasma membrane [2]. Strong interaction between intimin and Tir leads to an intimate attachment of EHEC to infected epithelial cells. Thirty distinct intimin subtypes with variation in their C-terminal part (involved in Tir binding) have been described so far [3] and this represents a standard tool for typing EHEC strains as well as for epidemiological studies. The biological significance of *eae* subtypes is not known but it has been suggested that intimin variants may be responsible for different host tissue cell tropisms [4]. The other cardinal virulence factor in EHEC is Stx, which is produced in the gut lumen and crosses the epithelial barrier to reach the bloodstream and the target organs including brain and kidneys. Stx binds speciﬁcally to globotriaosylceramide, a receptor present at the surface of glomerular endothelial cells, and blocks the translation in intoxicated cells resulting in cell death by apoptosis [5]. Two antigenically distinct forms of Stx can be produced by STEC, Stx1, and Stx2, and for each type of Stx up to seven subtypes have been described to date [6]. Stx2-producing *E. coli* strains, especially subtypes Stx2a and Stx2d, are more often associated with the most severe symptoms of the disease than Stx1-producing strains [7,8].

Because Stx induces bloody diarrhoea and renal dysfunction in infected patients, the toxin is considered as the main virulence factor of EHEC. However, many STEC strains isolated from the animal reservoir or from food products have never been associated with human infection and these strains have been classified as seropathotype E by Karmali and colleagues [9]. While several hypotheses could explain the occurrence of these putative non-pathogenic STEC strains (cryptic *stx* gene, undetected cases of human infection, weak EHEC survival during transmission to human, etc.), one hypothesis relies on the idea that Stx is necessary but not sufficient to cause disease in humans and that a minimal combination of virulence factors (including Stx) is required. In accordance with this hypothesis, epidemiological studies have emphasized that LEE is frequently associated with severe STEC infections [10,11]. However, about 10% of the confirmed STEC cases reported in the European Union between 2007 and 2010, including HUS patients, were caused by LEE-negative STEC strains [11]. These strains probably utilize other determinants to colonize the gut mucosa efficiently. Apart from intimin, EHEC produces many other potential adhesins or fimbriae that have been primarily described *in vitro* [12]. Similarly, many EHEC strains also carry large plasmids including the pO157 which encodes potential virulence factors such as the enterohaemolysin EhxA whose gene is used as a genetic marker for epidemiological studies [13]. However, the contribution of these determinants to the EHEC infectious process still needs to be evaluated. Over the past 15 y, the genome sequencing of many EHEC strains has provided new insights into their potential virulence determinants. In particular, the presence of additional DNA segments termed O-islands in the EHEC genome was linked to virulence potential since they contain several genes associated with virulence, including *stx*- and T3SS-encoding genes [14,15]. However, these O-islands also carry hundreds of genes of unknown predictive function that might be involved in EHEC infectious process. The pathophysiology of STEC infection in humans is therefore still far from being fully understood and more knowledge is needed to characterize the “auxiliary” factors that enable a STEC strain to cause disease in humans.

In this study, we applied a recombinase-based *in vivo* expression technology (RIVET) to the EHEC reference strain EDL933 in order to identify genes specifically induced during the infectious process, using mouse as an infection model. These *in vivo*-induced (*ivi*) genes would give us a subclass of genes encoding products potentially important for STEC pathogenesis. Thirty-one *ivi* genes have been identified and most of them are linked to general metabolism or to stress adaptive responses, or have poorly characterized functions. Among them, eight genes were not homogeneously distributed in *E. coli* and their prevalence was analysed in EHEC strains circulating in France these last years. Finally, the potential role of selected *ivi* genes in EHEC fitness has been investigated by competition assays in mice.

## Materials and methods

### Bacterial strains, plasmids, media and growth conditions

The bacterial strains and plasmids used in this study are listed in Table S1. The well-characterized O157:H7 EDL933 strain, which is *stx1*a^+^, *stx2*a^+^, and LEE^+^, was used throughout this study. The EDL933 mutants Δ*mhpR*, Δ*yhbU*, Δ*yftE*, Δ*yjiR*, ΔZ4799, ΔZ4070 and ΔZ3135 were constructed using the one-step PCR-based method [16]. Chromosomal deletion mutations were confirmed by PCR followed by DNA sequencing. The Δ*agaF* mutant was obtained using primers described in [17]. Bacteria were routinely grown in Luria-Bertani (LB) medium at 37°C unless otherwise indicated. For strain WM3064, 2,6-diaminopimelic acid (DAP) was added to a concentration of 100 µM. When required, antibiotics were used at the following concentrations: ampicillin (Amp), 50 µg.ml^−1^; kanamycin (Kan), 25 µg.ml^−1^; gentamicin (Gm), 15 µg.ml^−1^; chloramphenicol (Cm), 25 µg.ml^−1^; streptomycin (Sm), 50 µg.ml^−1^.

### Construction of an EHEC RIVET library

The EHEC RIVET library used in this study has been constructed using tools from the improved RIVET method described by Osorio *et al*. [18]. The strains used to construct the RIVET library, designated EDL-RES and EDL-RES1, are spontaneous streptomycin-resistant (Sm^R^) strains, harbouring a chromosomally located cassette, respectively, *res-kan-sacB-res* or *res1-kan-sacB-res1*. The strains were constructed by amplifying a cassette composed of *kan* and *sacB* genes and flanked by *res* sequences from plasmid pRES or pRES1 with primers RES-F (5ʹ-AAGCTGGAGCTCCACCGC-3ʹ) and RES-R (5ʹ- GATCGGGCCCACTATAGGGCGAATTGGGTACC-3ʹ). The PCR products were digested with *Sac*I and *Apa*I and inserted into the corresponding sites of pGP-Tn7-Gm. The corresponding constructs, pGP-Tn7-RES and pGP-Tn7-RES1, were then transferred to EDL933-Sm^R^ by mating experiments in order to integrate the *kan-sacB* cassette into the chromosome as described in [19]. Correct insertion of the cassette was validated by PCR and sequencing. Sucrose sensitivity of EDL-RES and EDL-RES1 strains was validated on LB plates without NaCl supplemented with 4% sucrose.

The EHEC RIVET library was constructed as follows. Genomic DNA from strain EDL933 was partially digested with *Sau*3AI. Fragments between 0.5 and 3 kb were gel-purified and cloned into the *Bgl*II site of vectors pGOA1193 or pGOA1195. The ligation mixtures were transformed into PIR1 strain. Transformants were selected on plates with Amp and pooled to perform a plasmid extraction en masse. The plasmid library was then transformed into WM3064, a strain used as the donor to mobilize plasmids into EDL-RES or EDL-RES1 by mating experiments, leading to plasmid integration into the EHEC genome due to its *ori*R6K origin of replication. Approximately 21,000 colonies, obtained on LB agar supplemented with Amp and Kan, were collectively resuspended and aliquots were frozen at – 80°C in 20% glycerol.

### *Screening of the RIVET library for* in vivo*-induced genes*

An aliquot of the RIVET library was grown 6 h at 37°C in LB with Amp and Kan. Bacteria were washed once in PBS and approximately 5.10^9^ CFU was intragastrically administered to 6-week-old BALB/c mice. After 18 h of infection, mice were euthanized and faeces or large intestine contents were collected and homogenized in sterile PBS. Serial dilutions were next spotted on LB agar + Sm or LB agar without NaCl + Sm and 4% sucrose. The plates were incubated for 16 h at 30°C. Individual sucrose-resistant colonies were tested for their sensitivity to Kan and resistance to Amp. Correct clones were grown in LB + Amp + Gm in 96-well plates and stored at – 80°C in 20% glycerol. DNA flanking the 5ʹ end of *tnpR* was amplified for each individual clone by SP-PCR [20] using the primer SP-PCR#1 (5ʹ-ATGACGTCACCTTCCTCCAC-3ʹ) and sequenced using the primer SP-PCR#2 (5ʹ-AGATGCGATTTGCTTTCACG-3ʹ). Obtained sequences were compared with the EHEC EDL933 genome using the MicroScope platform (https://www.genoscope.cns.fr/agc/microscope/home/index.php).

### *Construction of p*_chua_-tnpr *fusion strains*

EDL-RES and EDL-RES1 strains carrying *tnpR* gene under the control of *chuA* promoter region were constructed as follows. First, *chuA* promoter was amplified from genomic DNA of EDL933 using primers ChuA-F (5ʹ-GAAGATCTCGCAGGATAGGTTTCATAACC-3ʹ) and ChuA-R (5ʹ-GAAGATCTTAGCGATTCTCCATGAGGA-3ʹ), which contain *Bgl*II restriction sites in their 5ʹ ends. PCR product and vectors pGOA1193, pGOA1194 and pGOA1195 were digested with *Bgl*II and ligated separately to yield plasmids p1193-*chuA*, p1194-*chuA* and p1195-*chuA*. Correct orientation of *chuA* promoter was validated by PCR and sequencing. Plasmids were mobilized into EDL-RES and EDL-RES1 strains by mating experiments. Plasmid insertion into the *chuA* locus was verified by PCR and sequencing.

### Resolution assays

Excision of the RES marker cassette was monitored *in vitro* after growth in LB or *in vivo* after mouse infection. The percentage of bacteria that have lost the RES marker cassette, which is termed resolution, is calculated for an individual sample by dividing the titre on LB without NaCl agar plates with Sm and 4% sucrose by the titre on LB agar plates with Sm.

### Gene prevalence analyses

Distribution of *ivi* genes in 51 selected *E. coli* strains (listed in Table S2) was performed using the MicroScope platform (https://www.genoscope.cns.fr/agc/microscope/home/index.php) and completed when necessary using the BLAST program from the NCBI website. Then, distribution of eight *ivi* genes was evaluated on 228 selected strains obtained from the surveillance of EHEC infections from the French National Reference Centre for *Escherichia coli, Shigella* and *Salmonella*, Institut Pasteur, France (NRC). This collection represented all the 196 clinical EHEC strains received at NRC between 2015 and 2016 (n = 95 and 101, respectively, one clinical strain per patient) and was completed by food or environmental strains isolated in 2016 encompassing the serotype diversity. All the 32 non-human strains were isolated at the National Reference Laboratory, VetAgro Sup, France (LNR). Basic information as origin of isolates (human or food), year of isolation, clinical symptoms, and microbiological characteristics are shown in Table S3.

High-throughput whole-genome sequencing (WGS) was carried out on all *E. coli* strains at the “Plateforme de microbiologie mutualisée” (P2M) from the Pasteur International Bioresources network (Institut Pasteur, Paris, France). DNA extraction was carried out by using the MagNAPure 96 system (Roche). The libraries were prepared using the Nextera XT kit (Illumina) and the sequencing was done with the NextSeq 500 system (Illumina) generating 100 to 146 bp paired-end reads. Reads were trimmed and filtered using AlienTrimmer [21] with a quality Phred score threshold of 13 on a minimum length of 30 nucleotides. *De novo* assembly was performed with the SPAdes V.3.6.0 assembler [22]. Various genetic analyses were determined on SPAdes assembled sequences with web-based tools for WGS analysis developed by the Center for Genomic Epidemiology (CGE) (http://www.genomicepidemiology.org/). In particular, we searched O and H molecular serotyping, Multi-Locus-Sequence Typing (MLST) and main virulence gene content, i.e. *stx*1 and *stx*2 variants, *eae* variants, and *ehx*A. Furthermore, we extracted from each genome all the genes defining the phylogroup scheme [23]. A core-genome multi-alignment of all assembled genomes including an O157 *E. coli* reference genome (NC_002695) was done using the harvest v1.0.1 f ParSNP function [24]. The resulting single nucleotide polymorphisms (SNPs) were concatenated to generate a filtered multiple alignment that was used as input for the construction of a phylogenetic tree using MEGA6 [25], utilizing a maximum-likelihood (ML) approach. The final trees were visualized in the interactive Tree Of Life [26]. All genomes listed in supplementary Table S3 were uploaded on Enterobase (http://enterobase.warwick.ac.uk/species/ecoli) under the name indicated in “file NRC” item.

### Competition assays

Each of the *ivi* gene deletion strains was tested *in vivo* during competition assays in mice to evaluate if gene inactivation affects the bacterial fitness of EDL933 as previously described [27]. Briefly, individual EDL933 mutant and the wild-type (WT) strain were grown to mid-exponential phase in LB, washed once with PBS and mixed 1:1. Approximately 10^7^ CFU of both strains were administered intragastrically into 10 to 15 BALBc mice, 6 weeks of age, that were previously given drinking water containing Sm (5 g/L) for 24 h in order to selectively remove facultative anaerobic bacteria and open the niche for *E. coli* [27]. Eight days post-infection, faecal pellets were resuspended in PBS and plated on LB agar plates + Sm and LB agar plates + Sm + Kan to count WT + *ivi* mutant and *ivi* mutant alone, respectively. The WT population was obtained by subtracting CFU from LB + Sm + Kan plates to CFU from LB + Sm plates. Results are expressed as competitive indices obtained by dividing output ratio (WT/mutant day 8 post-infection) by the corresponding input ratio (WT/mutant from mouse inoculum).

### Statistical analyses

**Figure 1.**
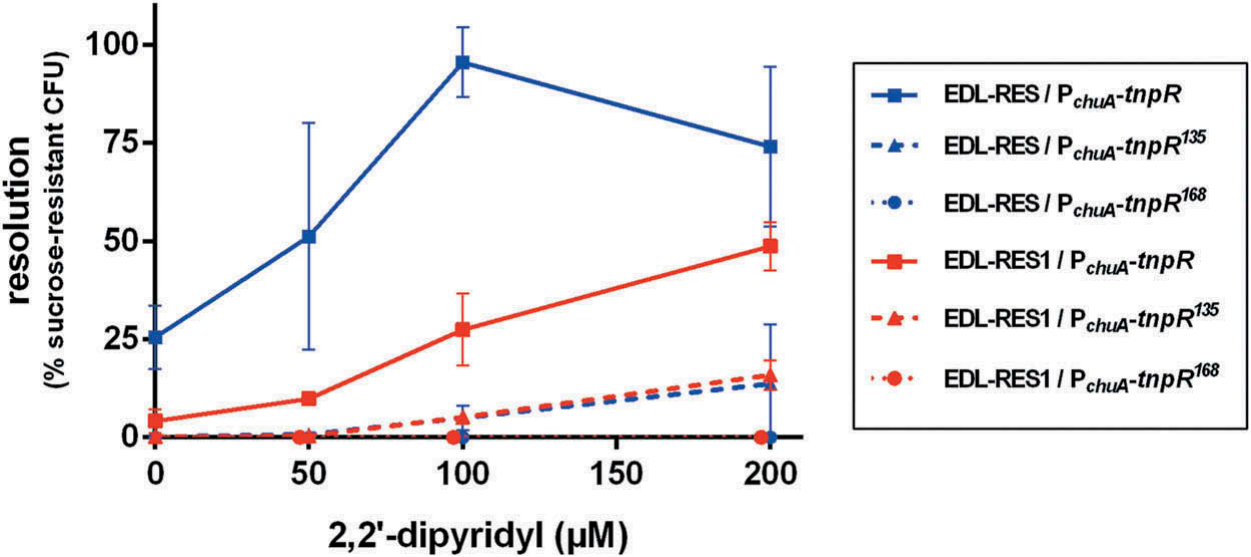
**Efficiency of the RIVET system in EHEC**. Indicated strains carrying *tnpR* gene under the control of *chuA* promoter were grown in LB supplemented with various concentrations of 2,2ʹ-dipyridyl. After 6 h of growth, cultures were enumerated on LB plates and on LB + sucrose plates to calculate the percentage of resolved CFU. Results are presented as mean and standard deviations from at least three independent experiments. Blue and red curves represent resolution obtained from the 3 *tnpR* variants inserted into EDL-RES and EDL-RES1 strains, respectively.

**Figure 2.**
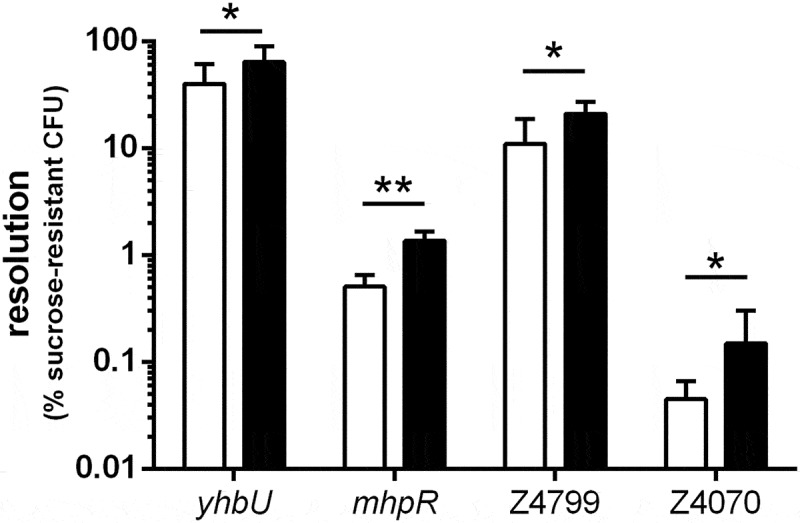
**Resolution of selected *ivi* genes following *in vitro* or *in vivo* growth**. EDL-RES strain carrying transcriptional fusion between indicated gene promoters and *tnpR* gene were either grown *in vitro* for 6 h in LB or were given to mice by oral gavage and feces were collected 16 h post-infection. Samples were then diluted and plated on LB plates or LB + sucrose plates to calculate the percentage of resolved CFU after *in vitro* growth (white bars) or following mouse challenge (black bars). Results are presented as mean and standard deviations from at least three independent experiments. Differences between *in vitro* and *in vivo* conditions were analyzed by a two-tailed unpaired t-test (*P < 0.05; **P < 0.01).

Clinical and microbiological data were entered anonymously. The Chi-square test or Fisher exact tests for proportions were performed. A *p*-value of <0.05 was considered significant. We used STATA version 12.0 (Stata Corporation, Texas, USA) for these analyses. Data in Figures 1 and 2 are presented as the mean ± the standard deviation of at least three independent experiments. Data in Figure 4 are presented as the median of two or three independent experiments depending on tested genes. ROUT method has been applied to exclude outliers. Student’s t-test or ANOVA with Fisher’s LSD test was used to determine significant differences between two or several test groups, respectively. We used PRISM version 6.01 (GraphPad software, Inc) for these analyses.

**Ethics Statement**. Animal experiments were reviewed and approved by the Auvergne Committee for Animal Experimentation (C2E2A). All procedures were carried out according to the European directives for the protection of animals used for scientific purposes, 2010/63/EU, and to the guidelines of the local ethics committee.

## Results

### Validation of the RIVET method in EHEC

To evaluate the efficiency of the RIVET method described by Osorio *et al*. [18] in EHEC, we placed the reporter gene *tnpR* under the control of the promoter region of *chuA*, for which expression is well known to be induced in iron starvation conditions [28]. Transcriptional fusions were made with three different alleles of *tnpR* (*tnpR, tnpR^135^, tnpR^168^*) and transferred to EDL-RES or EDL-RES1 strains that carry the RES or RES1 cassette carrying *sacB* and *kan* genes (see M&M), respectively (Table S1). These RES cassette variants and *tnpR* alleles have been conceived in order to detect a wider range of *ivi* genes through alteration of cassette excision and *tnpR* translation efficiencies, respectively [18]. Resolution (i.e. percentage of bacterial cells that have lost the RES cassette) was quantified after 6 h of growth in LB supplemented with various concentrations of the iron chelator 2,2ʹ-dipyridyl (Figure 1). In the presence of 2,2ʹ-dipyridyl, the level of resolution increased for each strain except for strains carrying the P*_chuA_-tnpR^168^* fusion, which were therefore not used thereafter. In addition, patterns of resolution were different between the strains. The strain most sensitive to iron starvation was EDL-RES carrying the P*_chuA_-tnpR* construct since it reached almost 100% resolution. Strain EDL-RES1 carrying the same construct was less sensitive to iron deficiency, with a maximal resolution of 49%. This result was expected since the RES1 cassette is excised by TnpR with a lower efficiency in comparison with the original RES cassette [18]. Both strains harbouring the P*_chuA_-tnpR^135^* construct had a low resolution that did not exceed 15%. Taken together, these results indicate that the RIVET strategy based on RES cassette – *tnpR* combination can be readily used in EHEC and that the use of different alleles of *tnpR* and RES cassettes may allow the identification of promoter regions with a broad range of promoter strength.

### Identification of *in vivo*-induced genes in EHEC using mouse as a host model

A genomic library of EHEC O157:H7 strain EDL933 was constructed by cloning random genomic DNA fragments upstream of *tnpR* genes (see M&M for details). More than 21,000 clones were obtained in EDL-RES or EDL-RES1 strains after selection on plates with kanamycin. Resistance to kanamycin implies that these clones still harbor the RES cassette and consequently do not carry an *in vitro* active promoter upstream of *tnpR*. The library was then inoculated intragastrically to conventional BALB/c mice. After 18 h of infection, bacteria were recovered from faeces either on LB with Sm to quantify the total number of EHEC or on LB with Sm and sucrose to select resolved strains. The percentage resolution after infection ranged from 0.01% to 6.75% between mice with a mean of 2.43%. We isolated a total of 1051 sucrose-resistant clones and 506 were confirmed to be sensitive to kanamycin. The DNA insert sequence was obtained from 335 isolates and 32 unique clones with integrated genes in the same orientation as *tnpR* were identified. Two clones correspond to the same gene (Z4799), thus leading to a list of 31 *in vivo*-induced (*ivi*) genes (Table 1). To validate our screening, we arbitrarily chose four genes and reconstructed transcriptional fusion with *tnpR* in EDL-RES strain. For each construct, resolution was recorded *in vitro* after growth in LB or *in vivo* after mice infection (Figure 2). For all strains, the resolution was higher following mouse infection than after *in vitro* growth, indicating that the expression level of each tested gene does indeed increase during the infectious process.

**Table 1.** List of *in vivo*-induced genes identified in EHEC O157:H7 strain EDL933.

	Gene			
COG category	label	name	O-island	Role or putative function
Metabolism				
[C]	*Z0072*	*araB*		L-Ribulokinase
[C]	*Z4726*	*nirB*		Large subunit of nitrite reductase
[E]	*Z3960*	*gabT*		4-Aminobutyrate aminotransferase
[E]	*Z3500*	*glpB*		*sn*-Glycerol-3-phosphate dehydrogenase
[E]	*Z2551*	*trpA*		α-Subunit of tryptophan synthase
[E]	*Z0607*	*ybaT*		Putative nitrogen-containing metabolite transporter
[E]	*Z5746*	*yjeH*		Member of the APC superfamily of amino acid transporters
[G]	*Z4486*	*agaW*	OI#126	Enzyme of the N-acetylgalactosamine phosphotransferase system
[H]	*Z2540*	*btuR*		Cobalamin adenolsyltransferase
[H]	*Z0061*	*pdxA*		4-Hydroxythreonine-4-phosphate dehydrogenase
[I]	*Z2315*	*azoR*		NADH-aZoreductase
[I]	*Z5366*	*fadA*		3-Ketoacyl-CoA thiolase
[P]	*Z5611*	*yjbB*		Putative transporter
Information storage and processing				
[J]	*Z4409*	*cca*		Multifunctional CCA protein
[K]	*Z0444*	*mhpR*		Transcriptional activator of 3-hydroxyphenylpropionic acid catabolism
[K]	*Z0073*	*araC*		Transcriptional regulator of L-arabinose transport and catabolism
[K]	*Z4022*	*ascG*		Transcriptional repressor of arbutin and salicin transport
[K], [E]	*Z5941*	*yjiR*		Putative transcriptional regulator
[K], [T]	*Z4017*	*norR*		Transcriptional activator of nitric oxide reductase NorV
[L], [U]	*Z4799*	-	OI#134	Putative DNA processing protein
Cellular processes and signaling				
[D]	*Z5820*	*ytfE*		Iron–sulphur cluster repair protein
[M]	*Z4886*	*yhiI*		Putative HlyD family secretion protein
[M]	*Z4926*	*mdtE*		Multidrug efflux system protein
[G], [E], [P], [R]	*Z5939*	*mdtM* (*yjiO*)		Multidrug efflux system protein
[O]	*Z4519*	*yhbU*		Putative collagenase
[O]	*Z4343*	*yghU*		Putative glutathione *S*-transferase
[O]	*Z4593*	*degQ*		Serine endoprotease
Poorly characterized				
[R]	*Z4070*	-	OI#111	Uncharacterized protein
[R]	*Z2757*	*ydjM*		Uncharacterized protein; regulated by the SOS system regulator LexA
[R]	*Z0964*	-	OI#36	Putative DNA packaging protein of prophage CP-933K
No COG				
-	*Z3135*	-	OI#80	Putative adhesin/invasin

COG category: [C] Energy production and conversion; [D] Cell cycle control, cell division, chromosome partitioning; [E] Amino acid transport and metabolism; [G] Carbohydrate transport and metabolism; [H] Coenzyme transport and metabolism; [I] Lipid transport and metabolism; [J] Translation, ribosomal structure and biogenesis; [K] Transcription; [L] Replication, recombination and repair; [M] Cell wall/membrane/envelope biogenesis; [O] Post-translational modification, protein turnover, and chaperones; [P] Inorganic ion transport and metabolism; [R] General function prediction only; [T] Signal transduction mechanisms; [U] Intracellular trafficking, secretion, and vesicular transport.

### Functional classification of *ivi* genes

Functional categories of the 31 *ivi* genes were assigned using the COG database [29,30]. Thirteen genes (42%) had a COG associated with the general category metabolism, seven (22.5%) with the category information storage and processing, seven (22.5%) with the category cellular processes and signalling, three (10%) with the category poorly characterized and one (3%) had no COG assignment (Table 1). A large number of *ivi* genes are involved in the metabolism of *i)* carbohydrates: L-arabinose (*araB, araC*) and N-acetyl-galactosamine (*agaW); ii)* amino acids: tryptophan (*trpA*), glutamate (*gabT*) and others (*yjeH); iii)* lipids: sn-glycerol-3P (*glpB*) and fatty acids (*fadA); iv)* vitamins: B_6_ (*pdxA*) and B_12_ (*btuR); v)* aromatic compounds: 3-hydroxyphenylpropionic acid (3HPP) (*mhpR*) and arbutin (*ascG*) or *vi)* nitrogen-containing metabolites (*nirB, ybaT*). The second largest class of *ivi* genes is related to stress resistance. Genes *mdtE* and *mdtM* encode subunits of multidrug efflux pumps. *yghU* encodes a glutathione S-transferase, and *yhiI* encodes a putative protein belonging to the MFP family, which is involved in the export of a variety of compounds including drugs. Genes *ytfE* and *norR* are related to nitrosative stress since it encodes, respectively, an iron-sulphur repair protein and a transcriptional activator of the *norVW* operon, whose products are involved in nitric oxide detoxification. There were also a number of *ivi* genes with unknown or poorly characterized function (*ydjM, yhbU*, Z0964, Z4070, Z4799, Z3135)

### Distribution of *ivi* genes in *E. coli*

We next analyzed *in silico* the distribution of *ivi* genes among 51 sequenced *E. coli* strains (Table S2). Twenty-three of the 31 *ivi* genes are highly conserved since they were detected in at least 98% of all strains. The eight other *ivi* genes present a prevalence ranging from 38% to 90% and half are located in O-islands [14] (Table 1 and S2). These genes encode transcriptional regulators (*mhpR, ascG* and *yjiR*), a protein from the Nle-encoding phage CP-933K (Z0964), a potential adhesin/invasin (Z3135), an efflux pump protein (*mdtM*), a putative DNA processing protein (Z4799) or an uncharacterized protein with a Cas3 domain (Z4070).

We next focused our attention on these eight *ivi* genes and analyzed their prevalence in the same *E. coli* collection after classification of strains as commensals, intestinal pathogens (InPEC) or extra-intestinal pathogens (ExPEC). As shown in Table 2, most of these *ivi* genes are not homogeneously distributed among the three categories. Interestingly, genes *mhpR, ascG, mdtM*, Z3135 and Z4070 present a higher prevalence in InPEC than in other groups (p < 0.03). Regarding the distribution of *ivi* genes in InPEC pathotypes, we observed that genes *yjiR* and Z3135 are more represented in EHEC than in other pathotypes (p < 0.01). Altogether, these data highlight a differential prevalence of several *ivi* genes in *E. coli*, with some of them being more represented within InPEC and/or EHEC groups, suggesting a potential role in the lifestyle of these pathogenic strains.

**Table 2.** Distribution of eight *ivi* genes in 51 *E. coli* strains grouped as commensals, InPEC or ExPEC.

	*mhpR*	*ascG*	*mdtM*	*yjiR*	Z0964	Z3135	Z4070	Z4799
commensal (n = 6)	0.83	0.83	0.83	0.67	0.33	0	0.33	0.33
InPEC (n = 31)	0.97	0.97	0.97	0.74	0.71	0.52	0.68	0.35
*EAEC (n = 3)*	*1*	*1*	*1*	*0.67*	*0.67*	*0*	*1*	*0*
*EHEC (n = 18)*	*1*	*1*	*1*	*1*	*0.78*	*0.72*	*0.72*	*0.5*
*EPEC (n = 4)*	*0.75*	*0.75*	*0.75*	*0.5*	*0.75*	*0.25*	*0.5*	*0.5*
*ETEC (n = 6)*	*1*	*1*	*1*	*0.17*	*0.5*	*0.33*	*0.5*	*0*
ExPEC (*n* = 14)	0.5	0.71	0.43	0.43	0.5	0.21	0.14	0.5

Values indicate the proportion of strains carrying *ivi* genes (columns) for each category (lines).

### Distribution of *ivi* genes in EHEC strains circulating in france

**Figure 3.**
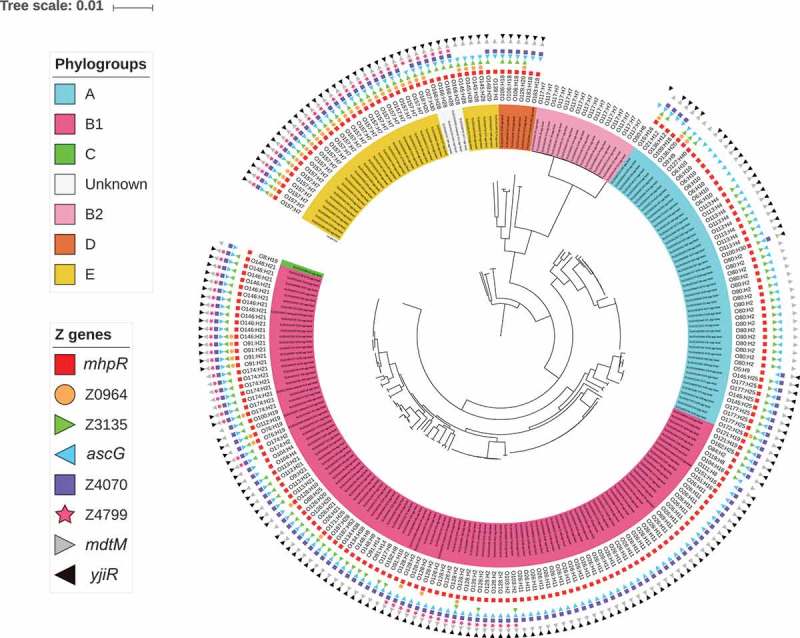
**Distribution of the eight *ivi* genes in the EHEC strain collection**. A phylogenetic tree was constructed after a core-genome multi-alignment with all 228 STEC strains and the O157 *E. coli* reference genome (NC_002695). Presence of each *ivi* gene is indicated by different coloured forms. Phylogroups are shown by colorizing the numbering of each strain and serotypes are written. All this information is indicated at the end of each branch of the phylogenetic tree.

To go further, we next evaluated the distribution of the eight *ivi* genes specifically in 228 EHEC strains isolated either from patients hospitalized in France between 2015 and 2016 or from contaminated foods (Table S3). The overall prevalence of the eight *ivi* genes ranges from 22% to 92%. First, we looked at the distribution of *ivi* genes in strains belonging to the 6 most commonly reported serotypes from severe cases of human STEC infections in the European Union [31] plus the emerging O80:H2 serotype [32,33] (defined here as the TOP seven priority serotype) versus other serotypes. We observed that all genes except Z3135 were more prevalent in strains from the TOP seven priority serotypes than in strains from other serotypes (Table 3; p < 0.003). Surprisingly, each *ivi* gene was almost exclusively present or absent in all strains from a given serotype (Table 3 and data not shown), suggesting a population-based distribution. For example, genes Z0964 and Z4799 were detected in all 27 O157:H7 strains but were not found in the genome of the 31 O26:H11 strains (Table 3). As the MLST types were concordant with the serotype, we found the same *ivi* gene distribution. Regarding their distribution by phylogroups (Table S4), some genes were more associated with a specific phylogroup than the others like genes Z0964 and Z4799 with phylogroup E and gene Z3135 with phylogroup A (p < 0.01). Distributions of the eight *ivi* genes according to serotype and phylogenetic population are shown in Figure 3.

**Table 3.** Distribution of the eight *ivi* genes in 228 EHEC strains classified by serotypes.

	*mhpR*	*ascG*	*mdtM*	*yjiR*	Z0964	Z3135	Z4070	Z4799
TOP 7 priority serotypes (n = 97)	1	1	1	0.82	0.37	0.66	0.77	0.46
*O157:H7 (n = 27)*	*1*	*1*	*1*	*1*	*1*	*0.96*	*0.96*	*1*
*O26:H11 (n = 31)*	*1*	*1*	*1*	*1*	*0*	*0*	*1*	*0*
*O103:H2 (n = 2)*	*1*	*1*	*1*	*1*	*0*	*0.5*	*1*	*1*
*O146:H21 (n = 12)*	*1*	*1*	*1*	*1*	*0.08*	*1*	*1*	*1*
*O91:H21 (n = 4)*	*1*	*1*	*1*	*1*	*1*	*1*	*1*	*1*
*O145:H28 (n = 4)*	*1*	*1*	*1*	*1*	*1*	*1*	*0*	*0*
*O80:H2 (n = 17)*	*1*	*1*	*1*	*0*	*0*	*1*	*0*	*0*
Other serotypes (*n* = 131)	0.86	0.87	0.86	0.77	0.11	0.58	0.72	0.24

Values indicate the proportion of strains carrying *ivi* genes (columns) for each category (lines).

We next evaluated the existence of a potential correlation between the presence of *ivi* genes and genes encoding characterized virulence factors. As shown in Table 4, all eight *ivi* genes were found to be more prevalent in strains harbouring *stx2* or *stx1*+ *stx2* than in strains carrying only *stx1* gene (P < 0.001). In contrast, only genes *mhpR, ascG, mdtM* and Z0964 were more prevalent in *eae*-positive strains than in *eae*-negative strains (P < 0.01). Interestingly, some *ivi* genes were found to be highly associated with particular intimin variants. For example, Z0964 was detected in all 31 strains carrying *eae* γ whereas it was only found in 1 of the 71 strains carrying other intimin variants (Table 4; P < 0.001). We finally investigated the distribution of *ivi* genes in strains for which we have clinical data in order to evaluate potential links between the presence of the 8 *ivi* genes in EHEC and severity of the induced disease. From our collection, 34 strains have been isolated from patients who have developed a HUS, 33 strains from patients with bloody diarrhoea and 47 strains from patients with watery diarrhoea. As expected, HUS-associated strains carry *stx2* and *eae* genes more frequently than strains causing diarrhoea only (Table 5, p < 0.01). In contrast, *stx1* is less prevalent in strains responsible for HUS. Regarding the eight *ivi* genes, no significant association was determined with clinical symptoms except for Z0964, which tends to be associated with HUS and/or bloody diarrhoea (P = 0.07).

**Table 4.** Co-distribution of the eight *ivi* genes with known virulence factors in the EHEC strain collection.

	*mhpR*	*ascG*	*mdtM*	*yjiR*	Z0964	Z3135	Z4070	Z4799
*stx1 + stx2* (n = 52)	1	1	1	1	0.37	0.71	0.92	0.67
*stx1a + stx2a (n = 13)*	*1*	*1*	*1*	*1*	*0.38*	*0.62*	*1*	*0.38*
*stx1a + stx2c (n = 7)*	*1*	*1*	*1*	*1*	*1*	*0.86*	*1*	*1*
*stx1a + stx2d (n = 7)*	*1*	*1*	*1*	*1*	*0.43*	*1*	*0.71*	*0.43*
*stx1c + stx2b (n = 23)*	*1*	*1*	*1*	*1*	*0.17*	*0.61*	*0.91*	*0.87*
*stx2* (n = 114)	0.99	1	0.99	0.76	0.23	0.75	0.75	0.3
*stx2a (n = 39)*	*1*	*1*	*1*	*0.92*	*0.31*	*0.51*	*0.9*	*0.28*
*stx2b (n = 12)*	*0.92*	*1*	*0.92*	*1*	*0.17*	*0.5*	*0.92*	*0.83*
*stx2c (n = 17)*	*1*	*1*	*1*	*0.88*	*0.41*	*1*	*0.82*	*0.47*
*stx2d (n = 28)*	*1*	*1*	*1*	*0.46*	*0.07*	*0.96*	*0.32*	*0*
*stx2e (n = 3)*	*1*	*1*	*1*	*0*	*0.33*	*0.33*	*0.67*	*0*
*stx2g (n = 3)*	*1*	*1*	*1*	*0.67*	*0.33*	*1*	*1*	*0*
*stx1* (n = 62)	0.73	0.73	0.73	0.68	0.1	0.27	0.56	0.11
*stx1a (n = 46)*	*0.63*	*0.63*	*0.63*	*0.61*	*0.07*	*0.17*	*0.59*	*0.07*
*stx1c (n = 15)*	*1*	*1*	*1*	*0.93*	*0.2*	*0.53*	*0.47*	*0.27*
*eae^+^* (*n* = 102)	1	1	1	0.82	0.31	0.62	0.77	0.28
*eae β* (*n* = 42)	*1*	*1*	*1*	*1*	*0*	*0.17*	*1*	*0*
*eae γ* (*n* = 31)	*1*	*1*	*1*	*1*	*1*	*0.97*	*0.84*	*0.87*
*eae ϵ* (*n* = 6)	*1*	*1*	*1*	*1*	*0.17*	*0.83*	*1*	*0.33*
*eae θ* (*n* = 3)	*1*	*1*	*1*	*0.67*	*0*	*0.33*	*0.67*	*0*
*eae ξ* (*n* = 17)	*1*	*1*	*1*	*0*	*0*	*1*	*0*	*0*
*eae^−^* (*n* = 126)	0.86	0.87	0.86	0.77	0.15	0.61	0.71	0.37

Values indicate the proportion of strains carrying *ivi* genes (columns) for each category (lines) NB: only categories with a strain number ≥3 are shown.

**Table 5.** Distribution of *stx, eae,* and the eight *ivi* genes in EHEC strains with regards to the symptoms they have caused in patients.

	*stx1*	*stx2*	*stx1* + *stx2*	*eae*	*mhpR*	*ascG*	*mdtM*	*yjiR*	Z0964	Z3135	Z4070	Z4799
*HUS* (*n* = 34)	0.03	0.79	0.18	0.71	1	1	1	0.85	0.29	0.71	0.85	0.32
*bloody diarrhoea* (*n* = 33)	0.15	0.55	0.3	0.49	0.94	0.94	0.94	0.82	0.3	0.61	0.85	0.42
*watery diarrhoea* (*n* = 47)	0.26	0.43	0.32	0.4	0.98	0.98	0.98	0.87	0.19	0.64	0.81	0.47

Values indicate the proportion of strains carrying indicated genes (columns) for each category (lines).

### Involvement of *ivi* genes in mouse gut colonization

**Figure 4.**
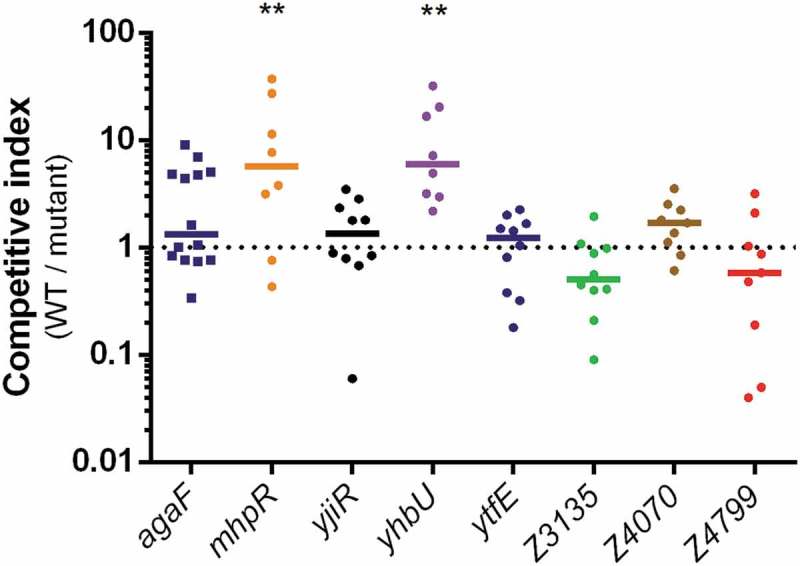
**Impact of *ivi* gene deletion on the fitness of EDL933**. Competition assays were performed by co-infecting mice with an equal mixture of EDL933 WT (Sm^R^) and the indicated *ivi* gene mutant (Sm^R^ Kan^R^). Eight days post-infection, faeces were sampled, homogenized in PBS, diluted and spotted on LB + Sm plates and LB + Sm + Kan plates to count, respectively, WT + *ivi* mutant and *ivi* mutant alone. The WT population was obtained by subtracting *ivi* mutant CFU from total EHEC CFU. Competitive indices (ratio WT/mutant) were calculated for each animal. Each dot represents one mouse and lines represent median values. Differences between each group and the control group *agaF* were analyzed by ANOVA with Fisher’s LSD test (** P < 0.01).

We next assessed the potential role of some *ivi* genes in the gut colonization process. Seven *ivi* genes have been arbitrarily selected based on their function potentially associated with bacterial fitness or virulence. This includes, for example, genes encoding putative adhesin or collagenase as well as genes encoding proteins involved in particular metabolic pathways. Of note, five of the seven selected genes belong to *ivi* genes heterogeneously distributed in *E. coli* and in EHEC strains as described above. We individually deleted the coding sequence of these seven *ivi* genes in EDL933 and tested each mutant by a competition assay with the WT strain during mouse infection (Figure 4). As a control, we also performed competitive infection using a strain deleted for *agaF*, which has been shown to be as efficient as the WT strain in colonizing mouse gut [34]. Deletion of genes *ytfE, yjiR*, Z3135, Z4070 or Z4799 has no impact on EDL933 colonization efficiency since calculated competitive indices between the WT strain and each mutant were closed to 1. In contrast, inactivation of *mhpR* or *yhbU* altered the ability of EDL933 to colonize the gut since both mutants were significantly outcompeted by the WT strain (Figure 4).

## Discussion

Since the first case of infection in the 1980s, the number of EHEC infections in humans has increased steadily and the pathogen is now recognized as a serious threat to human health. Stx and T3SS are the main virulence factors produced by EHEC strains, but the combinations of genetic traits that render a STEC strain highly virulent in humans are still poorly known. In this study, we performed RIVET screening using the EHEC O157:H7 reference strain EDL933 to identify genes that are induced *in vivo*, suggesting their potential role during the course of infection.

Among the 31 *ivi* genes identified, most are involved in adaptation of the pathogen to the gastrointestinal tract (GIT), as has been demonstrated in other studies [35,36]. First, genes involved in the metabolism of carbohydrates including arabinose (*araB, araC*), N-acetyl-galactosamine (*agaW*) and β-glucosides (*ascG*) have been detected as *ivi* genes. Proteins from these three pathways were also shown to be specifically expressed by EHEC in infected patients [35]. Interestingly, C-source metabolic EHEC profiling has shown that arabinose and N-acetylglucosamine could be relevant for identification of recent diversification events among serogroups and could be linked with functional specialization for the most hazardous EHEC strains [37]. Additionally, the utilization of arabinose (but not N-acetyl-galactosamine) confers an advantage on EHEC in efficient colonization of the mouse gut [34]. *ivi* Genes also include genes required for the metabolism of amino acids (tryptophan, glutamate), vitamins (B_6_, B_12_), fatty acids and aromatic compounds. For the latter category, expression of gene *mhpR* is induced during mouse infection. We also showed that the WT EDL933 strain has a competitive advantage over the *mhpR* mutant in colonizing mouse intestine. MhpR is the transcriptional activator of the *mhpABCDFE* operon that encodes enzymes dedicated to the transformation of 3HPP into intermediates of the Krebs cycle [38,39]. 3HPP is found naturally in the GIT of animals including humans since it results from anaerobic bacterial metabolism of plant-derived flavonoids or aromatic amino acids. Taken together with a recent study demonstrating that *mhp* gene expression in EDL933 is higher after growth in the caecal content of human gut microbiota-associated rats than after growth in the caecal contents of axenic rats [36], these data strongly suggest that EDL933 utilizes gut microbiota-produced 3HPP to efficiently colonize the GIT. It is noteworthy that oxygen is required for 3HPP catabolism by Mhp enzymes, and it has been suggested that *E. coli* performs these transformation steps close to epithelial cells where oxygen diffuses from blood [38].

In addition to metabolic adjustment, EHEC adapts to the GIT through the expression of genes involved in stress responses. Indeed, genes encoding components of the efflux pumps MdtEF and MdtM as well as a putative HlyD family secretion protein (YhiI) were found to be induced *in vivo*. These membrane structures are specialized in the export of a variety of harmful molecules such as drugs [40]. Other stress response pathways activated *in vivo* correspond to those involved in defence against nitrosative stress, with over-expression of the genes *mdtEF, ytfE*, and *norR*. The efflux pump MdtEF has been shown to protect *E. coli* against nitrosative stress during anaerobic respiration [41] and YtfE is an iron-sulphur cluster repair protein highly produced in the presence of nitric oxide (NO) through a direct regulation via the NO-sensing transcriptional regulator, NsrR [42,43]. NorR is a second NO-sensing transcriptional regulator that controls the expression of *norV* which encodes a NO reductase [42,43]. NorV by itself was identified as one of the *E. coli* O157:H7 proteins expressed during human infection [35]. Moreover, it has been shown that NorV plays an important role for NO detoxification under anaerobic conditions as well as for EHEC survival and Stx2 production within macrophages [44,45]. Authors of these works proposed *norV* as a direct virulence determinant for the pathogenesis of EHEC. We and others have also demonstrated that NO strongly affects the expression of both *stx* and LEE genes in EHEC, and therefore modulates the expression of key virulence factors [46–48]. Altogether, these data argue that establishment of an adapted response to nitrosative stress is a key component of both the adaptation of EHEC to the GIT and the control of its virulence program.

Despite the use of several *tnpR* and RES cassette variants to cover a broad range of promoter strength, genes encoding key virulence genes such as *stx* and LEE genes were not revealed in our screening. This finding has been already observed in other studies identifying *ivi* genes from different pathogens [18,49] and demonstrates that RIVET screenings do not provide an exhaustive list of fitness or virulence genes for a given pathogen. In addition to possible bias during construction of the library, identified *ivi* genes rely on their expression level during both *in vitro* and *in vivo* growth and can therefore be affected by experimental parameters (culture medium, animal model, infection route, bacterial inoculum dose, spatial and temporal sampling, etc.). Among these possibilities, absence of *stx* and LEE genes in our list of *ivi* genes might be explained by the fact that these genes are known to be expressed during growth in laboratory conditions and were probably discarded during the selection of inactive promoters *in vitro*. Nonetheless, several identified *ivi* genes are linked to processes known to affect the production of these two cardinal virulence factors. Indeed, gene *ydjM* is known to be part of the LexA regulon and its expression is highly induced following activation of the SOS system [50]. Strikingly, proteins encoded by the SOS-induced genes *uvrB* and *uvrY* have been detected as immunogenic O157:H7 proteins in HUS patients [35]. This converges towards an activation of the SOS response during infection and, as a consequence, induction of Stx phage lytic cycle and Stx2 production and release [51]. [46,47,52] Our list of *ivi* genes also includes *btuR* encoding a cobalamin adenosyltransferase involved in the assimilation of vitamin B_12_ [53] and *yjbB* encoding a transporter of inorganic phosphate [54]. Vitamin B_12_ and inorganic phosphate were both identified as molecules affecting the expression of Stx2 synthesis in EHEC [55,56].

Among the 31 identified *ivi* genes, 8 belong to the accessory genome as they were recovered in 22% to 93% of the 228 EHEC strains circulating in France between 2015 and 2016. Among these eight *ivi* genes, three (*mhpR, ascG,* and *mdtM*) were absent only in O117:H7 strains. Three other genes (*yjiR*, Z4799, and Z4070) presented a population-based distribution, suggesting gene acquisition by an ancestor and vertical transmission. Distribution of the last two genes (Z0964 and Z3135) is very heterogeneous among EHEC strains and suggests the occurrence of multiple acquisition events by different strains in the course of evolution. We also investigated the co-distribution of the eight *ivi* genes with known virulence genes. All *ivi* genes were more prevalent in strains harboring *stx2* (alone or associated with *stx1*) than in strains carrying only *stx1* gene (P < 0.001) and four of them (*mhpR, ascG, mdtM,* and Z0964) were more prevalent in *eae*-positive strains than in *eae*-negative strains (P < 0.01). Since epidemiological surveys determined that EHEC strains producing Stx2 and T3SS are more associated with the risk of HUS development [10], this raises the question whether one or more of these *ivi* genes contribute to the virulence potential of highly pathogenic EHEC strains. We were not able to make significant associations between *ivi* genes and clinical symptoms using our EHEC collection. However, this may be due to the paucity of clinical information collected in our study and future investigations need to be carried out to address this question. Among the eight *ivi* genes heterogeneously distributed in EHEC, three of them encode well-described or putative transcription factors (TF). As already discussed above, *mhpR* is the transcriptional activator of the *mhpABCDFE* operon involved in 3HPP catabolism. The second TF is AscG, a GalR-type TF which represses *ascFB* operon required for the transport and catabolism of arbutin, salicin, and cellobiose [57]. AscG has been shown to repress the *prpBCDE* operon involved in propionate catabolism [58]. Identification of the *mhpR* and *ascG* genes as *ivi* genes in our experiments suggests that EHEC would favour the utilization of 3HPP in the gut while limiting the utilization of arbutin, salicin, cellobiose, and propionate. The third TF, YjiR, encodes a putative transcriptional regulator of the MocR family as it possesses a clear Helix-Turn-Helix domain and an aspartate aminotransferase domain. While most regulators of the MocR family are still uncharacterized, the archetypal protein of this family, GabR, has been characterized to induce expression of the *gabTD* operon and thus to confer on *Bacillus subtilis* the ability to utilize γ -amino-butyric acid (GABA) as a nitrogen source [59]. While YjiR has only 24% identity to GabR, our RIVET screening also identified the gene *gabT*, which encodes GABA aminotransferase, suggesting the use of GABA by EDL933 in the GIT. However, co-infection assays demonstrated that deletion of *yjiR* does not alter the fitness of EDL933 in the GIT of infected mice. Nonetheless, all these data provide information on several substrates that could constitute specific nutrients used by EHEC to efficiently colonize the GIT. Several other genes belonging to the eight *ivi* genes heterogeneously distributed in EHEC can be correlated with virulence potential. First, gene Z0964 is located within prophage CP-933K that also includes genes *nleB, nleC, nleD* and *nleH1* encoding several known effectors of the T3SS [60–62]. Second, gene Z3135 encodes a large protein (2661 amino acids) of unknown function that contains multiple repeated domains with an Ig-like fold. These motifs are largely found in bacterial surface proteins belonging to the inverse autotransporter family such as intimins and invasins and are often involved in bacterial pathogenicity [63]. Finally, Z4799 encodes a putative DNA-processing protein belonging to a widespread bacterial protein family sharing a highly conserved domain called pfam02481. It is of interest that two proteins of this family, DprA from *Streptococcus pneumoniae* and Smf from *B. subtilis*, have been reported to play a prominent role in natural transformation through binding to exogenous single-stranded DNA and direct interaction with recombinase RecA [64]. Whereas *E. coli* is not a naturally transformable bacterial species, it is tempting to speculate that Z4799 can participate in activation of the SOS system in the case of DNA damage and thus can modulate the production level of Shiga toxins associated with RecA activation.

In this study, we performed genetic screening to identify genes from the EHEC O157:H7 strain EDL933 that are over-expressed *in vivo* during mouse infection. Among the 31 identified genes, a large proportion is involved in metabolic functions or in stress responses. The remaining *ivi* genes have hypothetic or unknown functions and several of them are potentially associated with EHEC virulence or fitness. We also demonstrated that eight *ivi* genes are heterogeneously distributed in EHEC strains circulating in France these last years. These genes are more prevalent in strains from the TOP seven priority serotypes, particularly strains carrying established virulence genes such as *stx* and *eae*. This work sheds further light on genes encoding niche factors that may increase the potential of STEC strains to cause disease in humans.
