# Supplementary material for: Identification and prevalence of in vivo-induced genes in enterohaemorrhagic Escherichia coli
Source: Virulence. 2019 Mar 16;10(1):180–93. doi: 10.1080/21505594.2019.1582976 (PMC6550539; doi:10.1080/21505594.2019.1582976)
Supplement: Supplemental Material [file kvir-10-01-1582976-s001.zip › Table S3.pdf]

file NRC	file NRL	ST (SLV_SingleLocusVariant)	Phylogroup	Molecular_serotype	Origin: Human Food	Year of isolation	symptoms when known	food source	stx1	stx2	eae	ehxA	*mhpR* (Z0444)	*ascG* (Z4022)	*mdtM* (Z5939)	*yjiR* (Z5941)	Z0964	Z3135	Z4070	Z4799
201500168		ST33	B1	O91:H14	H	2015			stx1a	stx2b	-	-	1	1	1	1		1	1	
201500207		ST724	E	O148:H20	H	2015	bloody diarrhea		stx1a	stx2d	-	ehxA	1	1	1	1		1	1	
201500209		ST10	A	O113:H4	H	2015			-	stx2c	-	-	1	1	1	1		1		
201500345		ST1049	B1	O128:H10	H	2015			stx1a	-	-	-	1	1	1	1	1		1	
201500346		ST69	D	O106:H18	H	2015	hemolytic uremic syndrom - death		-	stx2d	-	-	1	1	1	1		1	1	
201500347		ST448	B1	O148:H8	H	2015			-	stx2c	-	-	1	1	1	1		1	1	
201500350		ST10	A	O9:H9	H	2015			-	stx2e	-	-	1	1	1		1	1		
201500667		ST4748	B1	O128:H2	H	2015			stx1c	stx2b	-	-	1	1	1	1	1	1	1	1
201500734		ST32	E	O145:H28	H	2015			stx1a	-	eae γ	ehxA	1	1	1	1	1	1		
201501145		ST25	B1	O128:H2	H	2015			stx1c	stx2b	-	ehxA	1	1	1	1			1	1
201501170		ST11	E	O157:H7	H	2015			stx2c	-	eae γ	ehxA	1	1	1	1	1	1	1	1
201501365		ST504	B2	O117:H7	H	2015			stx1a	-	-	-								
201501723		ST21	B1	O26:H11	H	2015			stx1a	-	eae β	ehxA	1	1	1	1			1	
201501738		ST21	B1	O26:H11	H	2015			stx1a	-	eae β	ehxA	1	1	1	1			1	
201501835		ST25	B1	O128:H2	H	2015			stx1c	stx2b	-	ehxA	1	1	1	1			1	1
201501836		ST659	A	O145:H25	H	2015	diarrhea		stx2c	-	eae β	ehxA	1	1	1	1		1	1	
201501997		ST504	B2	O117:H7	H	2015			stx1a	-	-	-								
201502045		ST11	E	O157:H7	H	2015	bloody diarrhea		stx1a	stx2a	eae γ	ehxA	1	1	1	1	1	1	1	1
201502242		ST13	B1	O117:H8	H	2015			stx1c	-	-	ehxA	1	1	1	1			1	
201502308		ST5292 (SLV_ST504)	B2	O117:H7	H	2015			stx1a	-	-	-								
201502318		ST11	E	O157:H7	H	2015	bloody diarrhea		-	stx2a	eae γ	ehxA	1	1	1	1	1	1	1	1
201502340		ST659	A	O177:H25	H	2015			-	stx2d	eae β	ehxA	1	1	1	1		1	1	
201503031		ST5292 (SLV_ST504)	B2	O117:H7	H	2015	bloody diarrhea		stx1a	-	-	-								
201503097		ST56	B1	O113:H21	H	2015	acute renal failure		-	stx2d	-	-	1	1	1	1		1	1	
201503627		ST655	B1	O121:H19	H	2015	bloody diarrhea		-	stx2a	eae ε	ehxA	1	1	1	1		1	1	
201504139		ST141	B2	O50:H6	H	2015			-	stx2b	-	-		1		1				
201504401		ST739	B1	O9:H21	H	2015			-	stx2c	-	-	1	1	1	1		1	1	
201504668		ST504	B2	O117:H7	H	2015			stx1a	-	-	-								
201504709		ST504	B2	O117:H7	H	2015			stx1a	-	-	-								
201505035		ST301	A	O80:H2	H	2015	diarrhea		-	stx2d	eae ξ	ehxA	1	1	1			1		
201505176		ST11	E	O157:H7	H	2015	bloody diarrhea		stx1a	stx2c	eae γ	ehxA	1	1	1	1	1	1	1	1
201505530		ST11	E	O157:H7	H	2015			stx1a	stx2c	eae γ	ehxA	1	1	1	1	1	1	1	1
201505586		ST11	E	O157:H7	H	2015	bloody diarrhea		stx1a	stx2c	eae γ	ehxA	1	1	1	1	1	1	1	1
201505610		ST21	B1	O26:H11	H	2015			-	stx2a	eae β	ehxA	1	1	1	1			1	
201505920		ST11	E	O157:H7	H	2015			-	stx2c	eae γ	ehxA	1	1	1	1	1	1		1
201506018		ST11	E	O157:H7	H	2015	bloody diarrhea		stx1a	stx2c	eae γ	ehxA	1	1	1	1	1	1	1	1
201506067		ST11	E	O157:H7	H	2015	bloody diarrhea - thrombocytopenia		-	stx2c	eae γ	ehxA	1	1	1	1	1	1	1	1
201506143		ST32	E	O145:H28	H	2015			-	stx2d	eae γ	ehxA	1	1	1	1	1	1		
201506204		ST16	B1	O111:H8	H	2015			stx1a	-	eae θ	ehxA	1	1	1	1			1	
201506205		ST21	B1	O26:H11	H	2015			stx1a	-	eae β	ehxA	1	1	1	1			1	
201506298		ST21	B1	O26:H11	H	2015			-	stx2a	eae β	ehxA	1	1	1	1			1	
201506412		ST504	B2	O117:H7	H	2015			stx1a	-	-	-								
201506464		ST504	B2	O117:H7	H	2015			stx1a	-	-	-								
201506583		ST504	B2	O117:H7	H	2015			stx1a	-	-	-								
201506645		ST5292 (SLV_ST504)	B2	O117:H7	H	2015			stx1a	-	-	-								
201507423		ST297	B1	O171:H25	H	2015			stx2a	stx2d	-	ehxA	1	1	1	1	1	1	1	
201507462		ST11	E	O157:H7	H	2015	bloody diarrhea		-	stx2c	eae γ	ehxA	1	1	1	1	1	1	1	1
201507487		ST11	E	O157:H7	H	2015	bloody diarrhea - hemolytic uremic syndrom		-	stx2a	eae γ	ehxA	1	1	1	1	1	1	1	1
201508190		ST11	E	O157:H7	H	2015			stx1a	stx2c	eae γ	ehxA	1	1	1	1	1	1	1	1
201508381		ST21	B1	O26:H11	H	2015			stx1a	-	eae β	ehxA	1	1	1	1			1	
201508382		ST10	A	O127:H40	H	2015			stx1a	-	eae θ	-	1	1	1			1		
201508691		ST655	B1	O121:H19	H	2015	hemolytic uremic syndrom		-	stx2a	eae ε	ehxA	1	1	1	1		1	1	
201508692		ST21	B1	O151:H16	H	2015	bloody diarrhea		stx1a	-	eae β	ehxA	1	1	1	1			1	
201508989		ST21	B1	O26:H11	H	2015			stx1a	-	eae β	ehxA	1	1	1	1			1	
201509265		ST11	E	O157:H7	H	2015	hemolytic uremic syndrom		-	stx2a	eae γ	ehxA	1	1	1	1	1	1	1	1
201509622		ST32	E	O145:H28	H	2015	diarrhea		-	stx2c	eae γ	ehxA	1	1	1	1	1	1		
201509677		ST21	B1	O26:H11	H	2015	bloody diarrhea		stx1a	-	eae β	ehxA	1	1	1	1			1	
201509882		ST6060	E	O18:H20	H	2015			stx1c		-	-	1	1	1	1	1	1	1	1
201509986		ST641	B1	O91:H10	H	2015	hemolytic uremic syndrom		-	stx2c 1 stx2d	-	-	1	1	1	1		1	1	
201509987		ST17	B1	O103:H2	H	2015			stx1a	-	eae ε	ehxA	1	1	1	1		1	1	1
201510142		ST11	E	O157:H7	H	2015	hemolytic uremic syndrom		-	stx2a 1 stx2c	eae γ	ehxA	1	1	1	1	1	1	1	1
201510199		ST504	B2	O117:H7	H	2015			stx1a	-	-	-								
201510530		ST442	B1	O146:H21	H	2015	diarrhea		stx1c	stx2b	-	ehxA	1	1	1	1		1	1	1
201510531		ST442	B1	O146:H21	H	2015	diarrhea		stx1c	stx2b	-	ehxA	1	1	1	1		1	1	1
201510532		ST25	B1	O128:H2	H	2015	diarrhea		stx1c	stx2b	-	ehxA	1	1	1	1	1		1	1
201510533		ST56	B1	O113:H21	H	2015	hemolytic uremic syndrom		-	stx2c 1 stx2d	-	-	1	1	1	1		1	1	
201510534		ST398	A	O136:H20	H	2015	diarrhea		stx1c	-	-	-	1	1	1	1	1	1		
201510536		ST25	B1	O128:H2	H	2015			stx1c	stx2b	-	ehxA	1	1	1	1			1	1

201510537		ST21	B1	O69:H11	H	2015	bloody diarrhea		stx1a	-	eae β	ehxA	1	1	1	1			1	
201510538		ST753	E	O27:H30	H	2015	diarrhea		-	stx2b	-	-	1	1	1	1	1	1	1	1
201510539		ST1819	Unknown	O166:H28	H	2015			stx1c	-	-	ehxA	1	1	1	1		1	1	1
201510540		ST69	D	O106:H18	H	2015	bloody diarrhea		-	stx2d	-	-	1	1	1	1		1	1	
201510541		ST25	B1	O128:H2	H	2015	diarrhea		stx1c	stx2b	-	ehxA	1	1	1	1			1	1
201510542		ST675	B1	O76:H19	H	2015			stx1c	stx2b	-	ehxA	1	1	1	1	1	1	1	
201510543		ST442	B1	O146:H21	H	2015	diarrhea		stx1c	stx2b	-	ehxA	1	1	1	1		1	1	1
201510544		ST675	B1	O76:H19	H	2015			stx1c	-	-	ehxA	1	1	1	1	1	1	1	
201510545		ST10	A	O113:H4	H	2015			stx1c	stx2b	-	ehxA	1	1	1	1		1		
201510546		ST13	B1	O152:H8	H	2015			-	stx2b	-	-	1	1	1	1		1	1	
201510547		ST5296	B1	O112:H19	H	2015	diarrhea		-	stx2c	-	-	1	1	1	1		1	1	
201510548		ST677	B1	O174:H21	H	2015	diarrhea		-	stx2c 1 stx2d	-	-	1	1	1			1	1	1
201510549		ST329	A	O136:H12	H	2015	hemolytic uremic syndrom		-	stx2a	-	ehxA	1	1	1	1			1	
201510550		ST342	A	O5:H9	H	2015	diarrhea - hemolytic urmeic syndrom		stx1a	-	eae β	ehxA	1	1	1	1			1	
201510551		ST25	B1	O128:H2	H	2015	diarrhea		stx1c	stx2b	-	ehxA	1	1	1	1	1		1	1
201510552		ST25	B1	O128:H2	H	2015	bloody diarrhea		stx1c	stx2b	-	ehxA	1	1	1	1			1	1
201510554		ST21	B1	O151:H16	H	2015	diarrhea		stx1a	-	eae β	ehxA	1	1	1	1			1	
201510555		ST678	B1	O104:H4	H	2015			-	stx2a	-	-	1	1	1	1			1	
201510556		ST200	B1	O187:H28	H	2015	diarrhea		-	stx2g	-	ehxA	1	1	1	1		1	1	
201510557		ST442	B1	O146:H21	H	2015			-	stx2b	-	ehxA	1	1	1	1		1	1	1
201510558		Unknown ST	E	O8:H19	H	2015	bloody diarrhea		-	stx2e	-	-	1	1	1				1	
201510609		ST301	A	O80:H2	H	2015	hemolytic uremic syndrom		-	stx2d	eae ξ	ehxA	1	1	1			1		
201510618		ST32	E	O145:H28	H	2015	bloody diarrhea		-	stx2a	eae γ	ehxA	1	1	1	1	1	1		
201512012		ST301	A	O80:H2	H	2015			-	stx2d	eae ξ	ehxA	1	1	1			1		
201512308		ST677	B1	O174:H21	H	2015	bloody diarrhea		-	stx2c 1 stx2d	-	-	1	1	1	1		1	1	1
201512495		ST642	B1	O187:H52	H	2015			stx1c	-	-	-	1	1	1	1		1	1	
201513049		ST33	B1	O91:H14	H	2015	bloody diarrhea		stx1a	stx2b	-	ehxA	1	1	1	1		1	1	
201600250		ST21	B1	O26:H11	H	2016			stx1a	-	eae β	-	1	1	1	1			1	
201600750		ST25	B1	O128:H2	H	2016	diarrhea		-	stx2b	-	ehxA	1	1	1	1			1	1
201600751		ST25	B1	O128:H2	H	2016	bloody diarrhea		-	stx2b	-	ehxA	1	1	1	1			1	1
201600752		ST442	B1	O146:H21	H	2016			stx1c	stx2b	-	ehxA	1	1	1	1		1	1	1
201600753		ST442	B1	O146:H21	H	2016	diarrhea		stx1c	stx2b	-	ehxA	1	1	1	1		1	1	1
201600754		ST660	A	O172:H25	H	2016	hemolytic uremic syndrom		-	stx2a	eae ε	ehxA	1	1	1	1	1	1	1	
201600755		ST300	B1	O182:H25	H	2016			stx1a	-	eae ζ	ehxA	1	1	1	1		1	1	
201600757		ST678	B1	O104:H4	H	2016	bloody diarrhea		-	stx2a	-	-	1	1	1	1			1	
201600864		ST16	B1	O111:H8	H	2016			stx1a	-	eae θ	ehxA	1	1	1	1			1	
201600885		ST442	B1	O146:H21	H	2016			stx1c	stx2b	-	ehxA	1	1	1	1		1	1	1
201600976		ST677	B1	O174:H21	H	2016			-	stx2c	-	-	1	1	1			1	1	1
201601186		ST301	A	O80:H2	H	2016	bloody diarrhea - hemolytic uremic syndrom		-	stx2d	eae ξ	ehxA	1	1	1			1		
201601377		Unknown ST	B1	O91:H21	H	2016	bloody diarrhea - hemolytic uremic syndrom		stx1a	stx2d	-	ehxA	1	1	1	1	1	1	1	1
201601496		ST25	B1	O128:H2	H	2016			-	stx2b	-	ehxA	1	1	1	1			1	1
201601994		ST306	B1	O84:H2	H	2016			stx1a	-	eae ζ	ehxA	1	1	1	1		1	1	
201601997		ST504	B2	O117:H7	H	2016			stx1a	-	-	-								
201602029		ST11	E	O157:H7	H	2016	bloody diarrhea - hemolytic uremic syndrom		-	stx2a	eae γ	ehxA	1	1	1	1	1	1	1	1
201602147	03-17	ST154	B1	O134:H38	F	2016		frozen beef	stx1a	stx2a	-	ehxA	1	1	1	1		1	1	
201602148	19-45	ST677	B1	O174:H21	F	2016		frozen beef minced me	-	stx2c	-	-	1	1	1			1	1	1
201602149	03-18	ST329	A	O109:H16	F	2016		frozen beef minced me	-	stx2a	-	ehxA	1	1	1	1			1	
201602150	19-50	ST223	B1	O113:H21	F	2016		frozen beef	-	stx2a	-	ehxA	1	1	1	1		1	1	
201602151	29-37	ST10	A	O113:H4	F	2016		frozen beef	stx1a	stx2d	-	-	1	1	1	1		1		
201602152	29-38	ST10	A	O113:H4	F	2016		frozen beef minced me	stx1a	stx2d	-	-	1	1	1	1		1		
201602153	45-47	ST10	A	O113:H4	F	2016		frozen beef	-	stx2d	-	-	1	1	1	1		1		
201602154	49-31	ST223	B1	O113:H21	F	2016		frozen beef minced me	-	stx2a	-	ehxA	1	1	1	1		1	1	
201602155	49-51	Unknown ST	B1	O26:H21	F	2016		beef minced meat	-	stx2c 1 stx2d	-	-	1	1	1	1		1	1	
201602156	56-53	ST677	B1	O174:H21	F	2016		frozen beef minced me	-	stx2c 1 stx2d	-	-	1	1	1			1	1	1
201602157	56-55	ST677	B1	O174:H21	F	2016		frozen beef minced me	-	stx2c 1 stx2d	-	-	1	1	1			1	1	1
201602158	56-58	ST1794	B1	O26:H21	F	2016		beef minced meat	stx1a	stx2a	-	-	1	1	1	1		1	1	
201602159	61-16	ST58	B1	O88:H25	F	2016		frozen beef	stx1d	-	-	-	1	1	1			1	1	
201602161	72-49	ST325	A	O15:H16	F	2016		frozen beef	-	stx2g	-	-	1	1	1		1	1	1	
201602162	10-1	ST43	A	O6:H10	F	2016		goat cheese	stx1c	-	-	-	1	1	1	1				
201602163	10-4	ST43	A	O6:H10	F	2016		goat cheese	stx1c	-	-	-	1	1	1	1				
201602164	H12-42.1	ST43	A	O6:H10	F	2016		sheep cheese	stx1c	-	-	-	1	1	1	1				
201602165	H39-78	ST301	A	O80:H2	F	2016		raw milk cheese cow	-	stx2a	eae ξ	ehxA	1	1	1			1		
201602166	5576-1	ST442	B1	O146:H21	F	2016			stx1c	stx2b	-	ehxA	1	1	1	1		1	1	1
201602167	1635-121	ST1819	Unknown	O166:H28	F	2016		raw milk cheese cow	stx1c	-	-	-	1	1	1	1		1	1	1
201602168	2151-4	ST993	A	O100:H30	F	2016		raw milk cheese cow	-	stx2g	-	-	1	1	1	1		1	1	
201602169	ID12-42.2	ST43	A	O6:H10	F	2016		sheep cheese	stx1c	-	-	-	1	1	1	1				
201602170	H15-67.2	ST43	A	O6:H10	F	2016		raw milk cheese cow	-	stx2d	-	-	1	1	1	1				
201602172	4-3	ST677	B1	O174:H21	F	2016		raw milk cheese cow	-	stx2c	-	-	1	1	1	1	1	1	1	1
201602174	10-2	ST43	A	O6:H10	F	2016		goat cheese	stx1c	-	-	-	1	1	1	1				
201602175	H15-66.3	ST301	A	O80:H2	F	2016		raw milk cheese cow	-	stx2a	eae ξ	ehxA	1	1	1			1		
201602176	ID12-42.1	ST43	A	O6:H10	F	2016		sheep cheese	stx1c	-	-	-	1	1	1	1				

201602558		ST301	A	O80:H2	H	2016	hemolytic uremic syndrom		-	stx2d	eae ξ	ehxA	1	1	1			1		
201602633		ST504	B2	O117:H7	H	2016			stx1a	-	-	-								
201602747		ST301	A	O80:H2	H	2016	bloody diarrhea		-	stx2a	eae ξ	ehxA	1	1	1			1		
201602815		ST301	A	O80:H2	H	2016	hemolytic uremic syndrom		-	stx2d	eae ξ	ehxA	1	1	1			1		
201602954		ST504	B2	O117:H7	H	2016	diarrhea		stx1a	-	-	-								
201603424		ST21	B1	O26:H11	H	2016	diarrhea		stx1a	-	eae β	ehxA	1	1	1	1			1	
201603875		ST21	B1	O26:H11	H	2016	hemolytic uremic syndrom		-	stx2a	eae β	ehxA	1	1	1	1			1	
201604065		ST11	E	O157:H7	H	2016	diarrhea - hemolytic urmeic syndrom		-	stx2c	eae γ	ehxA	1	1	1	1	1	1	1	1
201604157		ST659	A	O177:H25	H	2016			-	stx2c	eae β	ehxA	1	1	1	1		1	1	
201604210		ST21	B1	O26:H11	H	2016	hemolytic uremic syndrom		-	stx2a	eae β	ehxA	1	1	1	1			1	
201604211		ST1611	B1	O100:H19	H	2016			-	stx2d	-	-	1	1	1			1	1	
201604763		ST661	B1	O174:H2	H	2016	hemolytic uremic syndrom		-	stx2a	-	ehxA	1	1	1	1		1	1	1
201604834		ST448	B1	O148:H8	H	2016	bloody diarrhea - hemolytic uremic syndrom		-	stx2c 1 stx2d	-	-	1	1	1	1		1	1	
201604931		ST301	A	O80:H2	H	2016			-	stx2d	eae ξ	ehxA	1	1	1			1		
201605072		ST5292 (SLV_ST504)	B2	O117:H7	H	2016			stx1a	-	-	-								
201605220		ST301	A	O80:H2	H	2016	diarrhea		-	stx2d	eae ξ	ehxA	1	1	1			1		
201605256		ST21	B1	O26:H11	H	2016	bloody diarrhea - hemolytic uremic syndrom		stx1a	stx2a	eae β	ehxA	1	1	1	1			1	
201605768	29F161036925-11	ST21	B1	O26:H11	F	2016		beef minced meat	stx1a	-	eae β	ehxA	1	1	1	1			1	
201605969		ST342	A	O145:H25	H	2016			-	stx2d	eae β	ehxA	1	1	1	1		1	1	
201606067		ST342	A	O145:H25	H	2016	bloody diarrhea		-	stx2c 1 stx2d	eae β	ehxA	1	1	1	1		1	1	
201606176		ST11	E	O157:H7	H	2016	hemolytic uremic syndrom		-	stx2a	eae γ	ehxA	1	1	1	1	1	1	1	1
201606625		ST21	B1	O26:H11	H	2016			-	stx2a	eae β	ehxA	1	1	1	1			1	
201606896		ST11	E	O157:H7	H	2016	diarrhea		stx1a	stx2c	eae γ	ehxA	1	1	1	1	1	1	1	1
201606918		ST607	A	O21:H12	H	2016	bloody diarrhea		-	stx2f	-	-	1	1	1	1		1	1	
201606919		ST301	A	O80:H2	H	2016			-	stx2d	eae ξ	ehxA	1	1	1			1		
201606956		ST10	A	O113:H4	H	2016	diarrhea		-	stx2d	-	-	1	1	1	1		1		
201607043		ST154	B1	O134:H38	H	2016			stx1a	stx2a	-	ehxA	1	1	1	1		1	1	
201607044		Unknown ST	B1	O91:H21	H	2016	hemolytic uremic syndrom		stx1a	stx2a	-	ehxA	1	1	1	1	1	1	1	1
201607045		ST301	A	O80:H2	H	2016			-	stx2d	eae ξ	ehxA	1	1	1			1		
201607153		Unknown ST	B1	O91:H21	H	2016	thrombotic microangiopathy		stx1a	stx2a	-	ehxA	1	1	1	1	1	1	1	1
201607651		ST657	D	O183:H18	H	2016			stx1a	stx2a	-	ehxA	1	1	1	1			1	
201607703		Unknown ST (SLV_ST659)	A	O177:H25	H	2016	diarrhea		-	stx2c	eae ε	ehxA	1	1	1	1		1	1	
201608481		ST11	E	O157:H7	H	2016	bloody diarrhea		-	stx2a	eae γ	ehxA	1	1	1	1	1	1	1	1
201608627		Unknown ST	B1	O91:H21	H	2016	bloody diarrhea - hemolytic uremic syndrom		stx1a	stx2d	-	ehxA	1	1	1	1	1	1	1	1
201608800		ST1967	B1	O103:H2	H	2016	diarrhea		stx1a	-	eae ε	ehxA	1	1	1	1			1	1
201609085		ST21	B1	O26:H11	H	2016	diarrhea		stx1a	-	eae β	ehxA	1	1	1	1			1	
201609162		ST11	E	O157:H7	H	2016	bloody diarrhea		stx1a	stx2c	eae γ	ehxA	1	1	1	1	1		1	1
201609174		ST21	B1	O26:H11	H	2016	diarrhea		stx1a	-	eae β	-	1	1	1	1			1	
201609293		ST21	B1	O26:H11	H	2016	hemolytic uremic syndrom		stx1a (50%)	stx2a	eae β	ehxA	1	1	1	1			1	
201609493		ST504	B2	O117:H7	H	2016			stx1a	-	-	-								
201609689		ST21	B1	O26:H11	H	2016	diarrhea		-	stx2a	eae β	ehxA	1	1	1	1			1	
201609690		ST21	B1	O26:H11	H	2016	diarrhea		-	stx2a	eae β	ehxA	1	1	1	1			1	
201609691		ST21	B1	O26:H11	H	2016	hemolytic uremic syndrom		-	stx2a	eae β	ehxA	1	1	1	1			1	
201609692		ST21	B1	O26:H11	H	2016	hemolytic uremic syndrom		-	stx2a	eae β	ehxA	1	1	1	1			1	
201609693		ST21	B1	O26:H11	H	2016			-	stx2a	eae β	ehxA	1	1	1	1			1	
201609694		ST21	B1	O26:H11	H	2016	diarrhea		-	stx2a	eae β	ehxA	1	1	1	1			1	
201609695		ST21	B1	O26:H11	H	2016	diarrhea		-	stx2a	eae β	ehxA	1	1	1	1			1	
201609696		ST21	B1	O26:H11	H	2016	hemolytic uremic syndrom		-	stx2a	eae β	ehxA	1	1	1	1			1	
201609720		ST1819	Unknown	O166:H28	H	2016	diarrhea		stx1c	-	-	ehxA	1	1	1	1		1	1	1
201609800		ST5292 (SLV_ST504)	B2	O117:H7	H	2016	bloody diarrhea		stx1a	-	-	-								
201609895		ST58	B1	O126:H20	H	2016			stx1a	-	-	-	1	1	1	1		1	1	
201609926		ST11	E	O157:H7	H	2016			-	stx2a	eae γ	ehxA	1	1	1	1	1	1	1	1
201609940		ST301	A	O80:H2	H	2016	diarrhea		-	stx2d	eae ξ	ehxA	1	1	1			1		
201609957		ST11	E	O157:H7	H	2016			stx1a	-	eae γ	ehxA	1	1	1	1	1	1	1	1
201610031		ST21	B1	O26:H11	F	2016		beef minced meat	stx1a	-	eae β	ehxA	1	1	1	1			1	
201610240		ST11	E	O157:H7	H	2016	diarrhea		-	stx2a	eae γ	ehxA	1	1	1	1	1	1	1	1
201610328		ST11	E	O157:H7	H	2016			-	stx2a	eae	ehxA	1	1	1	1	1	1	1	1
201610596		ST11	E	O157:H7	H	2016			-	stx2a	eae γ	ehxA	1	1	1	1	1	1	1	1
201610640		ST301	A	O80:H2	H	2016			-	stx2d	eae ξ	ehxA	1	1	1			1		
201610814		ST301	A	O80:H2	H	2016			-	stx2d	eae ξ	ehxA	1	1	1			1		
201610938		ST301	A	O80:H2	H	2016	hemolytic uremic syndrom		-	stx2d	eae ξ	ehxA	1	1	1			1		
201611058		ST11	E	O157:H7	H	2016			stx1a	stx2a	eae γ	ehxA	1	1	1	1	1	1	1	1
201611131		ST11	E	O157:H7	H	2016			stx1a	stx2a	eae γ	ehxA	1	1	1	1	1	1	1	1
201611225		ST25	B1	O128:H2	H	2016			stx1c	stx2b	-	ehxA	1	1	1	1			1	1
201611226		ST442	B1	O146:H21	H	2016	abdominal pain		-	stx2b	-	ehxA	1	1	1	1	1	1	1	1
201611227		ST661	B1	O174:H2	H	2016	bloody mucoid diarrhea		stx1a	stx2d	-	ehxA	1	1	1	1		1	1	1
201611228		ST25	B1	O128:H2	H	2016	abdominal pain		-	stx2b	-	ehxA	1	1	1	1			1	1
201611229		ST25	B1	O128:H2	H	2016	MAT		-	stx2b	-	ehxA	1	1	1	1			1	1
201611230		Unknown ST	B1	O104:H16	H	2016	diarrhea		stx1c	-	-	ehxA	1	1	1			1		
201611231		Unknown ST (SLV_ST25)	B1	O128:H2	H	2016	diarrhea		-	stx2b	-	ehxA	1	1	1	1		1	1	1
201611232		ST10	A	O113:H4	H	2016	diarrhea		stx1c	stx2b	-	ehxA	1	1	1	1		1		

201611233		ST1819	Unknown	O166:H28	H	2016	diarrhea		stx1c	stx2b	-	ehxA	1	1	1	1		1	1	1
201611236		ST342 (SLV_ST659)	A	O177:H25	H	2016	hemolytic uremic syndrom		-	stx2a/d	eae β	ehxA	1	1	1	1		1	1	
201611237		ST442	B1	O146:H21	H	2016	diarrhea		stx1c	stx2b	-	ehxA	1	1	1	1		1	1	1
201611238		ST442	B1	O146:H21	H	2016	diarrhea		-	stx2b	-	ehxA	1	1	1	1		1	1	1
201611239		ST58	B1	O126:H20	H	2016	pyelonephrite		stx1a	-	-	-	1	1	1	1		1	1	
201611240		ST25	B1	O128:H2	H	2016	hemolytic uremic syndrom		stx1c	stx2b	-	ehxA	1	1	1	1			1	1
201611241		ST69	D	O106:H18	H	2016	thrombotic microangiopathy		-	stx2d	-	-	1	1	1	1		1	1	
201611242		ST442	B1	O146:H21	H	2016	diarrhea		stx1c	stx2b	-	ehxA	1	1	1	1		1	1	1
201611243		SLV_ST657	D	O183:H18	H	2016	diarrhea		stx1a	stx2a	-	ehxA	1	1	1	1			1	
201611244		ST342 (SLV_ST659)	A	O177:H25	H	2016	hemolytic uremic syndrom		-	stx2c	eae β	ehxA	1	1	1	1		1	1	
201611245		ST10	A	O113:H4	H	2016	bloody mucoid diarrhea		-	stx2d	-	-	1	1	1	1		1		
201611248		ST1	E	O139:H1	H	2016	bloody mucoid diarrhea		-	stx2e	-	-	1	1	1				1	
201611249		ST590	B1	O119:H8	H	2016	hemolytic uremic syndrom		-	stx2d	eae α	-	1	1	1	1		1	1	
201611574		Unknown ST	D	O129:H20	H	2016			-	stx2d	-	-	1	1	1	1	1	1	1	
201611613	03H161D3211-62	ST21	B1	O26:H11	F	2016		beef minced meat	stx1a	stx2a	eae β	ehxA	1	1	1	1			1	
201611615	31H1611941401-A	ST21	B1	O26:H11	F	2016		raw milk cheese cow	-	stx2a	eae β	ehxA	1	1	1	1			1	
201611618	16I1611941553-A	ST21	B1	O26:H11	F	2016		raw milk cow	-	stx2a	eae β	ehxA	1	1	1	1			1	
201611816		ST21	B1	O26:H11	H	2016	diarrhea		stx1a	-	eae β	ehxA	1	1	1	1			1	
												**prevalence**	**92%**	**93%**	**92%**	**79%**	**22%**	**61%**	**74%**	**33%**
