# Supplementary material for: Identification and prevalence of in vivo-induced genes in enterohaemorrhagic Escherichia coli
Source: Virulence. 2019 Mar 16;10(1):180–93. doi: 10.1080/21505594.2019.1582976 (PMC6550539; doi:10.1080/21505594.2019.1582976)
Supplement: Supplemental Material [file kvir-10-01-1582976-s001.zip › Table S4new.docx]

**Table S4:** distribution of the 8 *ivi* genes in 228 EHEC strains classified by phylogroups

|  | *mhpR* | *ascG* | *mdtM* | *yjiR* | Z0964 | Z3135 | Z4070 | Z4799 |
| --- | --- | --- | --- | --- | --- | --- | --- | --- |
|  |  |  |  |  |  |  |  |  |
| Phylogroup A (n=50) | 1 | 1 | 1 | 0.60 | 0.08 | 0.80 | 0.30 | 0 |
| Phylogroup B1 (n=114) | 1 | 1 | 1 | 0.93 | 0.11 | 0.52 | 0.99 | 0.38 |
| Phylogroup B2 (n=18) | 0 | 0.06 | 0 | 0.06 | 0 | 0 | 0 | 0 |
| Phylogroup C (n=1) | ND | ND | ND | ND | ND | ND | ND | ND |
| Phylogroup D (n=6) | 1 | 1 | 1 | 1 | 0.17 | 0.67 | 1 | 0 |
| Phylogroup E (n=36) | 1 | 1 | 1 | 0.97 | 0.94 | 0.94 | 0.86 | 0.83 |
| Unknown (n=4) | 1 | 1 | 1 | 1 | 0 | 1 | 1 | 1 |

Values indicate the proportion of strains carrying *ivi* genes (columns) for each category (lines)

ND, not determined
