# Supplementary material for: Identification and prevalence of in vivo-induced genes in enterohaemorrhagic Escherichia coli
Source: Virulence. 2019 Mar 16;10(1):180–93. doi: 10.1080/21505594.2019.1582976 (PMC6550539; doi:10.1080/21505594.2019.1582976)
Supplement: Supplemental Material [file kvir-10-01-1582976-s001.zip › Table S1new.doc]

**Table S1:** Bacterial strains and plasmids used in this study.

| Strain or plasmid | Description | Source or reference |
| --- | --- | --- |
| **Strains** |  |  |
| EDL933 | Wild type EHEC O157:H7, *stx1*a^+^, *stx2*a^+^, LEE^+^ |  |
| EDL933-Sm^R^ | Streptomycin-resistant derivative of EDL933 | this work |
| EDL-RES | EDL933-Sm^R^ containing *res-kan-sacB-res* cassette, Sm^R^, Kan^R^, Suc^S^ | this work |
| EDL-RES1 | EDL933-Sm^R^ containing *res1-kan-sacB-res1* cassette, Sm^R^, Kan^R^, Suc^S^ | this work |
| EDL-RES / P*_chuA_*-*tnpR* | EDL-RES carrying a P*_chuA_*-*tnpR* fusion; Sm^R^ Kan^R^ Gm^R^ Amp^R^ | this work |
| EDL-RES / P*_chuA_*-*tnpR^168^* | EDL-RES carrying a P*_chuA_*-*tnpR*^mut168^ fusion; Sm^R^ Kan^R^ Gm^R^ Amp^R^ | this work |
| EDL-RES / P*_chuA_*-*tnpR^135^* | EDL-RES carrying a P*_chuA_*-*tnpR*^mut135^ fusion; Sm^R^ Kan^R^ Gm^R^ Amp^R^ | this work |
| EDL-RES1/ P*_chuA_*-*tnpR* | EDL-RES1 carrying a P*_chuA_*-*tnpR* fusion; Sm^R^ Kan^R^ Gm^R^ Amp^R^ | this work |
| EDL-RES1/ P*_chuA_*-*tnpR^168^* | EDL-RES1 carrying a P*_chuA_*-*tnpR*^mut168^ fusion; Sm^R^ Kan^R^ Gm^R^ Amp^R^ | this work |
| EDL-RES1/ P*_chuA_*-*tnpR^135^* | EDL-RES1 carrying a P*_chuA_*-*tnpR*^mut135^ fusion; Sm^R^ Kan^R^ Gm^R^ Amp^R^ | this work |
| PIR1 | F - ∆*lac*169 *rpoS*(Am) *robA*1 *creC*510 *hsdR*514 *endA recA*1 *uidA*(∆*Mlu*I)::*pir*-116 | Invitrogen |
| WM3064 | *thrB1*004 *pro thi rpsL hsdS lacZ*ΔM15 RP4-1360 Δ(*araBAD*)567 Δ*dapA*1341::[*erm pir*(wt)] |  |
| EDL933-Sm^R^ Δ*agaF* | EDL933-Sm^R^ Δ*agaF*, Kan^R^ | this work |
| EDL933-Sm^R^ Δ*Z3135* | EDL933-Sm^R^ Δ*Z3135*, Kan^R^ | this work |
| EDL933-Sm^R^ Δ*Z4519* | EDL933-Sm^R^ Δ*Z4519*, Kan^R^ | this work |
| EDL933-Sm^R^ Δ*Z5820* | EDL933-Sm^R^ Δ*Z5820*, Kan^R^ | this work |
| EDL933-Sm^R^ Δ*Z0444* | EDL933-Sm^R^ Δ*Z5820*, Kan^R^ | this work |
| EDL933-Sm^R^ Δ*Z5941* | EDL933-Sm^R^ Δ*Z5941*, Kan^R^ | this work |
| EDL933-Sm^R^ Δ*Z4070* | EDL933-Sm^R^ Δ*Z4070*, Kan^R^ | this work |
| EDL933-Sm^R^ Δ*Z4799* | EDL933-Sm^R^ Δ*Z4799*, Kan^R^ | this work |
|  |  |  |
| **Plasmids** |  |  |
| pGOA1193 | *oriR6K mobRP4 lacZ tnpR*; Ap^R^ |  |
| pGOA1194 | *oriR6K mobRP4 lacZ tnpR*^mut168^; Ap^R^ |  |
| pGOA1195 | *oriR6K mobRP4 lacZ tnpR*^mut135^; Ap^R^ |  |
| pRES | *oriR6K mobRP4 res-neo-sacB-res;* Ap^R^ Km^R^ |  |
| pRES1 | *oriR6K mobRP4 res1-neo-sacB-res1*; Ap^R^ Km^R^ |  |
| pGP-Tn7-Gm | *oriR6K mobRP4 Tn7-*Gm*;* Ap^R^ Gm^R^ |  |
| pGP-Tn7-RES | pGP-Tn7-Gm:: *res-kan-sacB-res* | this work |
| pGP-Tn7-RES1 | pGP-Tn7-Gm:: *res1-kan-sacB-res1* | this work |
| pSTNSK-Cm | *oriSC101*(Ts) *tnsABCD*; Km^R^, Cm^R^ |  |
| p1193-*chuA* | pGOA1193 P*_chuA_*-*tnpR*; Ap^R^ | this work |
| p1194-*chuA* | pGOA1194 P*_chuA_*-*tnpR^168^*; Ap^R^ | this work |
| p1195-*chuA* | pGOA1195 P*_chuA_*-*tnpR^135^*; Ap^R^ | this work |

References

1.	O'Brien AO, Lively TA, Chen ME, Rothman SW, Formal SB: ***Escherichia coli* O157:H7 strains associated with haemorrhagic colitis in the United States produce a *Shigella dysenteriae* 1 (SHIGA) like cytotoxin**. *Lancet* 1983, **1**(8326 Pt 1):702.

2.	Paulick A, Koerdt A, Lassak J, Huntley S, Wilms I, Narberhaus F, Thormann KM: **Two different stator systems drive a single polar flagellum in *Shewanella oneidensis* MR-1**. *Mol Microbiol* 2009, **71**(4):836-850.

3.	Osorio CG, Crawford JA, Michalski J, Martinez-Wilson H, Kaper JB, Camilli A: **Second-generation recombination-based *in vivo* expression technology for large-scale screening for *Vibrio cholerae* genes induced during infection of the mouse small intestine**. *Infect Immun* 2005, **73**(2):972-980.

4.	Crepin S, Harel J, Dozois CM: **Chromosomal complementation using Tn7 transposon vectors in *Enterobacteriaceae***. *Appl Environ Microbiol* 2012, **78**(17):6001-6008.
